# Low partner testing in high HIV prevalence setting in Freetown, Sierra Leone: a retrospective study

**DOI:** 10.1186/s13104-019-4662-9

**Published:** 2019-09-24

**Authors:** Sulaiman Lakoh, Emmanuel Firima, Darlinda F. Jiba, Momodu Sesay, Mariama Marco Conteh, Gibrilla Fadlu Deen

**Affiliations:** 1 Department of Internal Medicine, College of Medicine and Allied Health Sciences, University of Sierra Leone, Freetown, Sierra Leone; 2 Department of Medicine, University of Sierra Leone Teaching Hospitals Complex, Freetown, Sierra Leone; 3 Karolinska Institute, Stockholm, Sweden; 4 National HIV/AIDS Secretariat, Freetown, Sierra Leone; 5 National AIDS Control Programme, Freetown, Sierra Leone

**Keywords:** Connaught Hospital, Clients, Provider, Counselling

## Abstract

**Objective:**

Despite a 1.5% National HIV prevalence, less than 40% of people living with HIV in Sierra Leone know their status. Limited activities on testing partners of HIV patients could be contributory to this substantial unawareness of HIV status. We carried out a retrospective study aimed at assessing partner testing and HIV prevalence among adults (≥ 15 years) tested using Determine™ and SD Bioline as recorded in the HIV testing registers from January to December 2017 at Connaught Hospital, an urban tertiary hospital in Sierra Leone.

**Results:**

Of the 3808 clients tested for HIV, 2048 (53.8%) were females. The median age was 31 (IQR 24–42) years and 2104 (55.3%) were single. While 3014 (79.1%) had Provider-Initiated Testing and Counseling (PITC), 794 (20.9%) had Client-Initiated Testing and Counseling (CITC). HIV test was positive in 925 (24.3%) {CI 22.9–25.6, P < 0.001} clients. Of the 17 (0.4%) partners tested for HIV, 9 (52.9%) were positive. PITC yielded more HIV positive cases (760, 25.2%) than CITC (165, 20.8%). Partner testing (P = 0.007), female sex (P < 0.001) and PITC (P = 0.006) were associated with a positive HIV diagnosis. With high HIV prevalence and low partner testing, activities on partner testing are needed to improve the response to the epidemic.

## Introduction

Despite the remarkable global success in reducing its associated morbidity and mortality [1], HIV remains a public health concern in Sierra Leone. Even though the reported country-wide adult HIV prevalence is 1.5% [2], less than 40% of people living with HIV (PLHIV) know their status [2]. Studies on HIV prevalence in healthcare settings are limited in the country. One health facility-based study reported an HIV prevalence of 8.9% among febrile patients in Southern Sierra Leone [3].

Although described as a heterogeneous epidemic with a dominant heterosexual transmission [4], the focus of HIV prevention and testing interventions in Sierra Leone is on key populations (men who have sex with men, female sex workers, etc.) [5], to the disadvantage of the general population attending health facilities.

The National AIDS Control Programme works to access more people with its HIV interventions, towards meeting the Joint United Nations Programme on HIV and AIDS (UNAIDS) 90-90-90 target [6]. To achieve this, PLHIV must first be tested [6], emphasizing the importance of developing innovative testing approaches and strengthening existing HIV testing and prevention services [7]. HIV testing is needed to cascade treatment, care, and support services for positive patients, and strengthen prevention services [8].

HIV testing through voluntary assisted partner notification is one of the novel approaches recommended by the World Health Organization (WHO) with the aim to improve access to partner testing [8]. Several studies enumerate the benefits of partner testing. Apart from expanding the diagnosis of HIV and increasing access to prevention, care and treatment services, partner testing improves adherence and retention in treatment, and where serodiscordant, help to prioritize effective prevention services, immediate ART commencement and adherence, as well as pre-exposure prophylaxis for seronegative partners [9–11]. Despite the recommendations and multiple evidence on new approaches to partner and couple testing, its practice has not been widely implemented and incorporated into HIV intervention programmes, although some African countries are employing the traditional passive partner notification approach where individuals testing positive for HIV are encouraged to bring their partners for testing [7].

Sierra Leone, like other African countries, is implementing the passive partner notification approach in order to improve access to partner testing, but there has been minimal impact due to poor compliance caused by fear of stigmatization [12]. An innovative approach to partner notification and testing is therefore required to improve access to HIV testing services, especially in high prevalence settings.

The study aimed to assess the state of partner testing as an approach for expansion of testing and improvement of diagnosis of PLHIV in a large urban tertiary hospital in Freetown and explored the prevalence of HIV at this centre using HIV testing data.

## Main text

### Methodology

A retrospective cross-sectional study design was employed to extract data for 3808 adults (≥ 15 years) clients tested using the WHO guiding principles [13] with third-generation Determine™ and SD Bioline kits as recorded in the HIV testing registers from January to December 2017 at Connaught Hospital, an urban tertiary hospital with the largest HIV clinic in Sierra Leone.

All extracted data were entered into Microsoft excel sheet secured with a password, transferred and analysed using SPSS version 21 and R. Data were summarised using proportions (categorical data) and median and interquartile ranges (non-normally distributed continuous data). Chi-square was used to carry out a bivariate analysis. Binomial logistic regression was used to determine the predictors of outcome variables.

A few clients with incomplete data were excluded from the study. Pregnant women were also excluded from the study as they were not tested in this facility.

#### Ethical issues

Ethical approval was obtained from the Sierra Leone Ethics and Scientific Review Committee of the Ministry of Health and Sanitation. To ensure confidentiality, the data was entered by the staff of the HIV centre and stored anonymously.

#### Definition of terms [13]

While CITC is a form of HIV testing services in which the patients initiate the process of counselling and testing, PITC denotes HIV testing services that are routinely offered in health facilities.

### Results

#### Characteristics of the clients

Of the 3808 clients tested for HIV, the majority (3014, 79.1%) were tested as part of PITC, while 794 (20.9%) were tested as CITC. Most (2048, 53.8%) clients were females with a median age of 31 years (IQR 24–42). The median age was 29 years (IQR 23–39.5) for females and 34 years (IQR 26–45) for males. Over half (2104, 55.3%) of the clients were single. Table 1 summarizes the main characteristics of the clients.

**Table 1:** Prevalence of HIV infection by patient characteristics

Variable	**N**	HIV positive n (%)	95% CI	P value	NNS N/n
Total	3808	925 (24.3)	22.9–25.6		4.1
Entry point				< 0.001	
PITC	3014	760 (25.2)	23.7–27.8		4.0
CITC	794	165 (20.8)	17.9–23.6		4.8
Age (years)				< 0.001	
15 and below	73	10 (13.7)	5.6–21.8		7.3
16 to 25	1088	191 (17.6)	15.3–19.8		5.7
25 to 35	1195	329 (27.5)	25.0–30.1		3.6
36 to 49	854	265 (31.0)	27.9–34.1		3.2
50 and above	598	130 (21.7)	18.4–25.1		4.6
Sex				< 0.001	
Male	1760	368 (20.9)	19.0–22.8		4.8
Female	2048	556 (27.1)	25.2–29.1		3.7
Marital status				0.730	
Single	2104	508 (24.1)	22.3–26.0		4.1
Married	1664	406 (24.4)	22.3–26.5		4.1
Widowed	39	11 (28.2)	13.4–43.0		3.5
Partner tested				0.001	
Yes	17	9 (52.9)	26.5–79.4		1.9
No	3791	916 (24.2)	22.8–25.5		4.1

#### Client testing

Of the 3014 clients tested under PITC, 1568 (51.9%) were females. Likewise, more females (485, 61.1%) voluntarily tested under CITC. Most (899, 29.8%) of the clients under PITC belonged to the 26–35 age group, whereas 301 (38%) clients for CITC belonged to the 16–25 age group. Of all females tested, 696 (34%) belonged to the 16–25 age group, whereas of all (568, 32.3%) males tested belonged to the 26–35 age group. Most (31/39) widowed clients and 10 (58.8%) of 17 partners tested had testing under PITC. Table 1 summarizes testing by entry point and sex.

#### HIV prevalence and Number needed to screen (NNS)

Of the clients, 925 (24.3%, CI 22.9–25.6, P < 0.001) were found to be HIV positive, giving an HIV prevalence of 24.3% in this population. Of the clients tested, only 17 (0.4%) of their partners had an HIV test with most (9, 52.9%) being HIV positive. The median age at diagnosis of HIV was 33 years. Additional file 1: Figure S1 shows the median age for clients by sex and marital status.

**Fig. 1:**
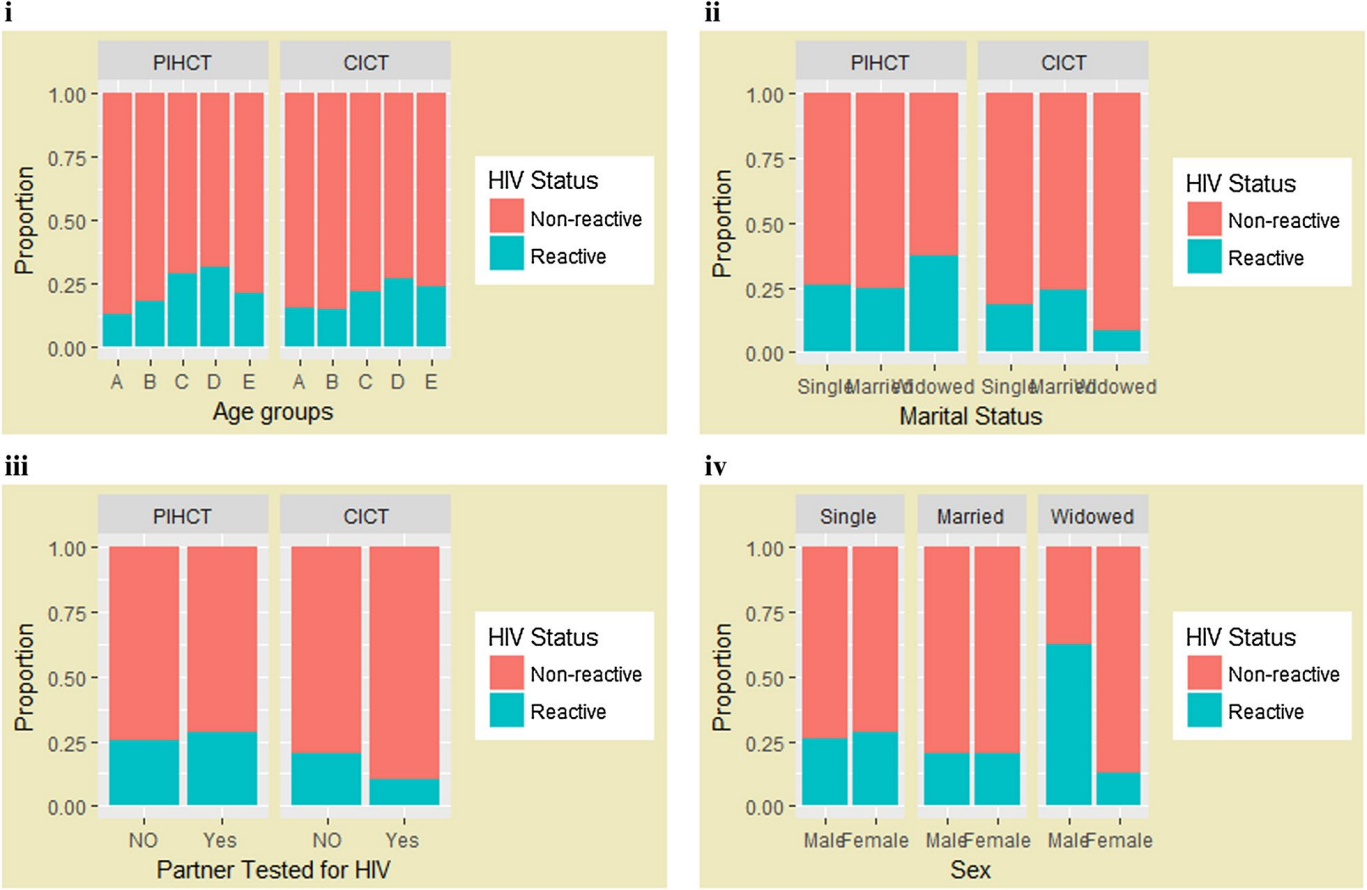
**i** Proportion of HIV positive patients by age groups, disaggregated by testing under PITC and CITC. A = 15 years and below; B = 16–25 years; C = 26–35 years; D = 36–49 years; E = 50 years and above. **ii** Proportion of HIV patients by marital status segregated by PITC and CITC. **iii** Proportion of HIV patients by partner tested, segregated by PITC and CITC. **iv** Proportion of patients with reactive HIV results, segregated by sex and marital status

Testing under PITC yielded more HIV positive cases (760, 25.2%), relative to positivity under CITC (165, 20.8%). A similar picture was observed when data were segregated by age group, marital status and partner tested (Fig. 1i–iii). The 36–49-year age group had the majority of positive cases (265, 31%) relative to other age groups. Of the females tested, 556 (27.1%) were HIV positive. Eleven (28.2%) of widowed patients were found to be HIV positive; 63% of widowed males were HIV positive compared to widowed females who had a prevalence of 13% (Fig. 1iv).

The NNS to diagnose one case of HIV was 4.1. Among those aged 36–49 years, NNS was 3.2. The NNS to diagnose one female HIV patient was 3.7 compared to 4.8 for males. Among widowed patients, NNS was 3.5. Regarding tested partners, NNS to diagnose one HIV patient was 1.9.

#### Predictors of HIV

Table 2 shows the result of multivariable logistic regression analysis. Following adjustments for potential confounders, it was found that relative to those 50 years and above, clients 25 years and below were less likely to be positive for HIV: 15 years and below (aOR = 0.47, CI 0.23–0.96, P = 0.038); 16 to 25 years (aOR = 0.68, CI 0.49–0.85, P = 0.002). Similarly, married clients were less likely to be HIV positive (aOR = 0.79, CI 0.66–0.93, P = 0.006).

**Table 2:** Multivariable analysis of factors associated with HIV infection

Variable	Adjusted OR (95% CI)	P value
Entry point		
PITC	1.32 (1.08–1.61)	0.006
CITC	1	
Age (years)		
15 and below	0.47 (0.23–0.96)	0.038
16 to 25	0.64 (0.49–0.85)	0.002
26 to 35	1.29 (1.01–1.64)	0.043
36 to 49	1.59 (1.25–2.04)	< 0.001
50 and above	1	
Sex		
Male	1	
Female	1.5 (1.32–1.80)	< 0.001
Marital status		
Single	1	
Married	0.79 (0.66–0.93)	0.006
Widowed	0.85 (0.41–1.75)	0.655
Partner tested		
No	1	
Yes	3.84 (1.45–10.20)	0.007

The age groups 26–35 (aOR = 1.29, CI 1.01–1.64) and 36–49 (aOR = 1.59, CI 1.25–2.04), females (aOR = 1.5, CI 1.32–1.80) and testing under PITC (aOR = 1.32, CI 1.08–1.61) were significantly associated with a positive diagnosis of HIV. Partner testing was nearly 4 times more likely to yield a positive HIV patient (aOR = 3.8, CI 1.45–10.2, P = 0.007).

### Discussion

As there is no previous published study on partner testing of HIV in Sierra Leone, we assessed partner testing of clients tested for HIV at Connaught Hospital and determine the HIV prevalence among this population.

Our study has demonstrated low partner testing of clients tested for HIV in a high prevalence setting. The proportion of clients tested for HIV who correspondingly had their partners tested was extremely low, estimated at 0.4%. Limited disclosure of HIV status to sexual partners could be a major explanation for this low prevalence of partner testing.

Undisclosed HIV status to sexual partners is a huge barrier to HIV testing services in low- and middle-income countries (LMICs) [14]. Several studies have identified low disclosure of HIV status to sexual partners in developing countries [15, 16]. There are many barriers to the disclosure of HIV status observed by various studies in LMICs. Illiteracy, stigmatization, male sex of index client, abandonment, fear of accusation for infidelity and limited knowledge about HIV are among the barriers to non-disclosure of HIV status in these studies [12, 17, 18].

Addressing issues of stigma, discrimination and gender disparity will improve uptake of HIV testing services [19] and position developing countries to achieve the UNAIDS ambitious global target of 90-90-90 [1].

In a systematic review of partner notification of HIV status, WHO observed increased uptake of HIV testing services with assisted partner notification [20]. WHO recommends the implementation of assisted partner notification as a measure to improve uptake of HIV testing [20].

The overall prevalence of HIV among people tested in this facility was 24.3%, making Connaught Hospital a high HIV prevalence setting [13]. This is above the national prevalence of 1.5% [21, 22] and 8.9% among febrile patients in a secondary hospital in Sierra Leone [3]. The modes of HIV transmission study in Sierra Leone observed higher prevalence among key populations relative to the general population. HIV prevalence in these groups was highest in transgender male to female (22.4%), followed by men who have sex with men (14.0%), people who inject drugs (8.5%) and female sex workers (6.7%) [23]. Even though these groups are vulnerable and at higher risk of acquiring HIV [24], it is obvious that the prevalence in any one of them was lower than the observed prevalence in patients tested under PITC.

With relatively low partner testing in this hospital population coupled with the high likelihood of yielding a positive result among the few tested partners, reprogramming of targeted HIV testing services is needed. Individuals presented to health facilities and their sexual partners should be considered a high priority group for HIV testing services. WHO and UNAIDS have recommended HIV self-testing, and testing through voluntary assisted partner notification as new approaches to increase testing uptake [8].

Evidence from many countries and different population support the importance of HIV self-testing. HIV self-testing provides an opportunity for increasing uptake of services and to reach out to hidden populations [8]. Self-testing also provides a confidential atmosphere for individuals and a platform for stigma reduction [25]. As a cost-effective intervention, HIV self-testing has been recommended for implementation in sub-Saharan Africa [26].

Patients tested under PITC were more likely to have an HIV positive result compared to patients tested under CICT (25.2% vs 20.8%). Evidence from multiple studies in LMICs has shown the effectiveness of PITC as a measure to increase HIV case detection and testing rate [27, 28]. WHO and UNAIDS recommend HIV testing and counselling by healthcare providers to all adults and adolescents in all health facilities [29].

In conclusion, we demonstrated a high HIV prevalence and low prevalence of partner testing at Sierra Leone’s main tertiary hospital where over 50% chance of a positive HIV test was observed among tested couples.

To increase partner testing, stakeholders in the HIV response should address issues of disclosure and expand on existing HIV testing strategies like PITC to all health facilities in Sierra Leone with a specific focus on partner testing. Self-testing and other new HIV testing approaches can be adopted together with enhanced partner notification and testing.

## Limitations

Our study has several limitations. It is a single site study in an urban tertiary hospital where a high HIV prevalence is expected. Although a national referral hospital, its findings may not be representative of the general situation of HIV testing and prevalence in Sierra Leone.

Again, the study utilized secondary data with limited variables impairing further analysis of participants’ characteristics including sexual orientation, substance abuse and current status of sexual partner. Nonetheless, the findings on this large cohort of clients are a concern for the prevention and control of the HIV epidemic in Sierra Leone and are expected to inform policies on the HIV response.

## Supplementary information

**Additional file 1: Figure S1.** Median age at HIV diagnosis by i. Marital status, and ii. Sex.

## Data Availability

Data is available at 10.6084/m9.figshare.8636894.v1.

## Abbreviations

AIDS: acquired immunodeficiency syndrome
CITC: Client Initiated Testing and Counselling
HIV: human immunodeficiency virus
LMICs: low- and middle-income countries
NNS: number needed to screen
PICT: Provider Initiated Testing and Counselling
PLHIV: people living with HIV
UNAIDS: United Nations Programme on HIV/AIDS
WHO: World Health Organization
