# Supplementary figures and images for: Low partner testing in high HIV prevalence setting in Freetown, Sierra Leone: a retrospective study
Source: BMC Res Notes. 2019 Sep 24;12:629. doi: 10.1186/s13104-019-4662-9 (PMC6760048; doi:10.1186/s13104-019-4662-9)

i ii


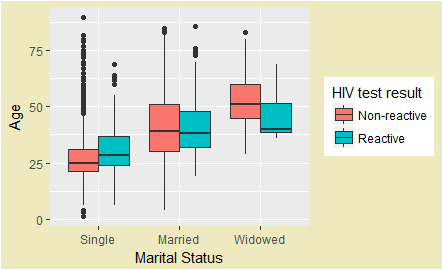

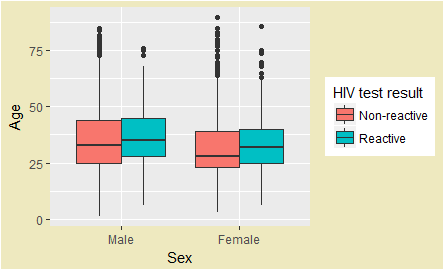


**Figure S1. Median age at HIV diagnosis by i. Marital status, and ii. Sex.**

Supplement: Supplementary file 1 — Additional file 1: Figure S1. Median age at HIV diagnosis by i. Marital status, and ii. Sex. [file 13104_2019_4662_MOESM1_ESM.docx]
